# Supplementary material for: P-TRAP: a Panicle Trait Phenotyping tool
Source: BMC Plant Biol. 2013 Aug 29;13:122. doi: 10.1186/1471-2229-13-122 (PMC3848748; doi:10.1186/1471-2229-13-122)
Supplement: Additional file 7 — P-TRAP source code. The Netbeans project that contains the source code of the application. [file 1471-2229-13-122-S7.zip › javadoc.zip!/javadoc/org-basmati-workspace/org/basmati/workspace/CollectMasterReportAction.html]

CollectMasterReportAction (WorkSpace)


JavaScript is disabled on your browser.


- Package
- Class
- Tree
- Deprecated
- Index
- Help

*org.basmati.workspace 1.0*

- Prev Class
- Next Class

- Frames
- No Frames

- All Classes

- Summary:
- Nested |
- Field |
- Constr |
- Method

- Detail:
- Field |
- Constr |
- Method


org.basmati.workspace

## Class CollectMasterReportAction

- java.lang.Object
- - org.basmati.workspace.CollectMasterReportAction

- All Implemented Interfaces:
  :   java.awt.event.ActionListener, java.util.EventListener

  ---

    

  ```
  public final class CollectMasterReportAction
  extends java.lang.Object
  implements java.awt.event.ActionListener
  ```

  The action handler when the user click on the collect report button.

- - ### Constructor Summary

    Constructors

    Constructor and Description

    `CollectMasterReportAction(org.bioalg.riceprojecttype.RiceProject context)`
  - ### Method Summary

    Methods

    | Modifier and Type | Method and Description |
    | --- | --- |
    | `void` | `actionPerformed(java.awt.event.ActionEvent ev)` |
    | `org.openide.filesystems.FileObject[][]` | `mapStructureGrainsFiles(org.openide.filesystems.FileObject[] files)` Given a set of grain and structure result files, this function maps them to a (n/2)\*2 matrix of structure (in the first column) and grain (in the second column). |

    - ### Methods inherited from class java.lang.Object

      `clone, equals, finalize, getClass, hashCode, notify, notifyAll, toString, wait, wait, wait`

- - ### Constructor Detail


    - #### CollectMasterReportAction

      ```
      public CollectMasterReportAction(org.bioalg.riceprojecttype.RiceProject context)
      ```
  - ### Method Detail


    - #### actionPerformed

      ```
      public void actionPerformed(java.awt.event.ActionEvent ev)
      ```

      **Specified by:**
      :   `actionPerformed` in interface `java.awt.event.ActionListener`


    - #### mapStructureGrainsFiles

      ```
      public org.openide.filesystems.FileObject[][] mapStructureGrainsFiles(org.openide.filesystems.FileObject[] files)
      ```

      Given a set of grain and structure result files, this function maps them to a (n/2)\*2 matrix of structure (in the first column) and grain (in the second column).

      Parameters:
      :   `files` - an array of files

      Returns:
      :   a 2D matrix maps the input files

      See Also:
      :   `FileObject`


- Package
- Class
- Tree
- Deprecated
- Index
- Help

*org.basmati.workspace 1.0*

- Prev Class
- Next Class

- Frames
- No Frames

- All Classes

- Summary:
- Nested |
- Field |
- Constr |
- Method

- Detail:
- Field |
- Constr |
- Method
